# Different cultures and sexual unwellness in older adults: A qualitative study with older populations from Slovenia and Portugal

**DOI:** 10.1192/j.eurpsy.2022.373

**Published:** 2022-09-01

**Authors:** S. Von Humboldt, J. Rolo, J.A. Ribeiro-Gonçalves, E. Benko, G. Low, I. Leal

**Affiliations:** 1 ISPA – Instituto Universitário, Lisbon, Portugal, William James Research Center, Lisbon, Portugal; 2 Instituto Universitário, Ispa, Lisbon, Portugal; 3 University of Primorska, Faculty Of Health Sciences, Izola, Slovenia; 4 University of Alberta, Faculty Of Nursing, Edmonton, Canada

**Keywords:** Qualitative study, Cultural diversity, sexual unwellness, Older Adults

## Abstract

**Introduction:**

Oder adults have been stereotyped (1), both explicitly and implicitly, as being asexual or naturally lacking sexual desires (2).

**Objectives:**

The objective of this study is to analyse the perspectives of sexual unwellness (SU) of Portuguese and Slovenian older adults.

**Methods:**

A qualitative research was carried out, in which these perceptions were analysed at a cultural level. Methods: The sample of this study consisted of 136 older participants, between 65 and 96 years of age. Participants were of two different nationalities and lived in the community. Participants were interviewed, and all interviews were carried out through the process of literal transcription and subsequent content analysis.

**Results:**

Eight key mutually exclusive themes emerged from the interviews: unavailability of partner; traditional values; body restrictions; low self-esteem and well-being; poor social support; dissatisfaction with physical appearance; pain during sex; and difficulties meeting new people. Unavailability of partner was the most important theme (17.9%) for the studied sample and specifically among Portuguese participants. Conversely, difficulties meeting new people was the least reported theme (6.8%) for the entire sample. For Slovenians traditional values were most relevant with respect to feeling sexually unwell.

**Conclusions:**

Older adults from two different countries reported diverse sexual experiences. Eight mutual-exclusive themes were extensively illustrated. 1.von Humboldt S et al. Sexual expression in old age: How older adults from different cultures express sexually? Sex Res Social Policy. 2020;1-15. 2.von Humboldt S et al. Are older adults satisfied with their sexuality? Outcomes from a cross-cultural study. Educ Gerontol. 2020;46:284-293.

**Disclosure:**

No significant relationships.

